# “Reassurance that you're doing okay, or guidance if you're not”: A qualitative descriptive study of pregnant first time mothers’ expectations and information needs about postnatal care in England

**DOI:** 10.1016/j.midw.2020.102813

**Published:** 2020-10

**Authors:** Jenny McLeish, Merryl Harvey, Maggie Redshaw, Fiona Alderdice

**Affiliations:** a NIHR Policy Research Unit in Maternal and Neonatal Health and Care, National Perinatal Epidemiology Unit, University of Oxford, Old Road Campus, Headington, Oxford OX3 7LF, UK; b School of Nursing and Midwifery, City South Campus, Birmingham City University, Westbourne Road, Birmingham B15 3TN, UK

**Keywords:** Postnatal care, Information, Expectations, Qualitative, Pregnant women, Primiparous

## Abstract

**Objective:**

To explore what first time mothers in England expect from postnatal care while they are pregnant, what they would ideally like, where they get their information on postnatal care, and their views on the sufficiency of this information.

**Design:**

A qualitative descriptive interview-based study.

**Setting:**

England

**Participants:**

A maximum variation sample of 40 women who were currently in the third trimester of pregnancy; aged 16 or over; planning to give birth in England and had not given birth previously.

**Methods:**

Semi structured interviews were carried out between October 2017 and March 2018, by telephone (*n* = 32) and face to face (*n* = 8). Interviews were analysed using thematic analysis.

**Results:**

There were six themes and twelve subthemes. The themes were: (1) ‘Piecing together snippets of information’ containing subthemes ‘Incomplete official sources’ and ‘Other mothers’ stories’; (2) ‘Planning ahead or going with the flow’ containing subthemes ‘Wanting more information’ and ‘Postnatal care not a priority’; (3) ‘Judgement or reassurance’ containing subthemes ‘Real: Being judged’, ‘Ideal: Reassurance and non-judgmental advice’; (4) ‘Focus of care’ containing subthemes ‘Real: A focus on checks and feeding’, ‘Ideal: More focus on mother's wellbeing’; (5) ‘A system under pressure’ containing subthemes ‘Real: Busy midwives, reactive care’, ‘Ideal: Reliable, proactive information’; (6) ‘Deciding about discharge’, containing subthemes ‘Real: Confusion about decision-making’, ‘Ideal: More control over length of hospital stay’.

**Key conclusions:**

Fi**r**st time mothers’ experience of the transition to parenthood could be improved by antenatal access to comprehensive information about the timing, location, content and purpose of postnatal care. Information should take a woman-centred perspective and cover all settings (hospitals, birth centres, home, community), including the roles and responsibilities of all the professionals who may be involved.

**Implications for practice:**

Clear and comprehensive information about postnatal care should be provided to all women in ways that are accessible at any stage of pregnancy or the postnatal period. As women pregnant for the first time worry about being judged if they seek professional advice and reassurance postnatally, information about postnatal care should aim to address this.

## Introduction

Understanding expectations of healthcare can contribute to quality improvement and identify the information about care that people may need to be realistically prepared (Kravitz, 1996). Little is known about what pregnant women expect from postnatal care or how well informed they feel in advance about postnatal care. Although national guidance in England directs that every woman should have an individualised postnatal care plan, ideally drawn up in pregnancy and regularly reviewed (Demott et al., 2006; National Maternity Review, 2016), this guidance is not consistently followed (Newburn and Bhavnani, 2019; Royal College of Midwives, 2014).

Expectations are a multi-dimensional concept; for example, Thompson and Suñol (1995) distinguish four types: ideal (preferences), predicted (real, anticipated outcomes), normative (what ought to happen) and unformed (where the person does not have any clear expectations). A small amount of research has explored pregnant women's *ideal* expectations for postnatal care in Australia, England and Sweden. These included: staying in hospital after birth until they felt confident to leave; practical care, guidance and information from staff, including home visits and telephone support in the community; socialisation with visitors and other mothers in hospital; and rest (Forster et al., 2008; Hirst and Hewison, 2002; Lindberg et al., 2008). The only *real* expectations about postnatal care reported in these studies were pregnant women's fears that they would not be able to stay in hospital as long as they wanted (Forster et al., 2008; Hirst and Hewison, 2002). Primiparous pregnant women may have different *ideal* expectations to multiparous women due to their different needs (Forster et al., 2008; Lindberg et al., 2008). They are also likely to have different *real* expectations, as multiparous mothers’ expectations will be influenced by their own previous experiences, whereas first time mothers are more reliant on external sources of information (Ockleford et al., 2004).

Other research has considered ‘expectations’ of postnatal care retrospectively, after birth. Some studies have inferred *ideal* or *normative* expectations from what new mothers felt was missing from the postnatal care they had actually received, such as that staff should be kinder, more attentive, offer more support, and give consistent information (Beake et al., 2005; Fawcett, 2016; Jomeen and Redshaw, 2013; Ockleford et al., 2004; Puthussery et al., 2010; Redshaw and Henderson, 2012). It is not reported whether any of these expectations were also held during pregnancy. Some studies give limited information about what new mothers said retrospectively they had *really* expected when pregnant, such as a busy ward environment, low quality food, and staff taking the baby so the mother can rest (Beake et al., 2010; Bowling et al., 2012; Forster et al., 2008; Puthussery et al., 2010). The reliability of expectations described after an event has, however, been questioned, as they may be misremembered or adjusted in the light of outcomes (Thompson and Suñol, 1995).

In order to support first time mothers’ transition to parenthood by more effectively meeting their needs for information about the postnatal care that will be available, it is therefore important to understand their current expectations of postnatal care and information needs, by exploring these prospectively and distinguishing between real and ideal expectations. Taking into consideration the limitations of current research, this paper aims to explore what first time mothers really expect from postnatal care while they are pregnant, what they would ideally like, where they get their information on postnatal care, and whether they feel that the information they have is sufficient. It reports research that is part of a programme of work on first time mothers’ expectations and experiences of postnatal care that includes an online survey and a qualitative longitudinal study, which will be reported separately.

## Methods

### Design and ethical approval

This was a qualitative descriptive interview-based study (Sandelowski, 2000). The ‘low inference’ design (Sandelowski, 2000) was chosen because the purpose of the study was to explore participants’ own expectations and information needs while pregnant, and thus to stay close to their perspectives without imposing a theoretical framework or generating theory (Tindall, 1994). At the same time, this study acknowledges the roles of both participants’ understandings and the researchers’ interpretations in the production of knowledge (Madill et al., 2000; Sandelowski, 2010). The University of Oxford Medical Sciences Inter-Divisional Research Ethics Committee (reference R52703/RE001) approved the study.

### Setting/recruitment

The study was carried out in England. Criteria for participation were that women were currently in the third trimester of pregnancy; aged 16 or over; planning to give birth in England and had not given birth previously. Purposive maximum variation sampling (Patton, 2002) was used to recruit women with a range of socio-demographic characteristics. Multiple recruitment strategies were used to include women who are less likely to participate in research (Bonevski et al., 2014) and in particular younger women and women living in more deprived areas, who are less likely to respond to postal and online maternity surveys (Redshaw and Henderson, 2015). These were: [1] An invitation at the end of an online survey about expectations of postnatal care, promoted on social media (Facebook and Twitter) by parenting organisations – the results of this survey will be reported separately; [2] An in-person invitation from a researcher to women attending three sessions of a young mothers’ antenatal group and two sessions of a free antenatal exercise class, each run by a community group in a different area of high deprivation; [3] An advertisement circulated on social media by a multiple birth charity. The researchers had no prior contact with any of the participants.

### Data collection

Data were collected through semi-structured qualitative interviews between October 2017 and March 2018. Women who responded to the survey invitation or advertisement were telephoned or emailed to explain the purpose of the research. They were emailed a participant information sheet at least 24 h before the interview, which was carried out by telephone. Where there was face-to-face contact, the researcher explained the purpose of the research, gave women a participant information sheet and offered the options to take part in a face-to-face interview at a time and place of their choice, or a telephone interview.

Interviews were structured around three subject areas: information about postnatal care, real expectations of postnatal care and ideal expectations. Topics included the source of the interviewee's ideas on postnatal care, what she wanted to know about postnatal care when she was pregnant, what she expected it would be like staying in the hospital or birth centre after birth, the help she expected from staff and how they would interact with her, the type of postnatal care she expected in the community, and the postnatal care she would like in an ideal world. The topic guide for the interviews is available as Supplementary File A.

Informed consent was obtained at the beginning of the interview through a signed consent form if face-to-face, or given orally and recorded in writing if by telephone. Participants were offered a shopping voucher worth £10 at the end of the interview to thank them for their time. No one else was present during interviews, which were carried out in English, audio-recorded and fully professionally transcribed. Each participant was given an anonymous identifier beginning with PNC for ‘postnatal care’ and personally identifying details were removed from transcripts.

In longitudinal qualitative research it is usual to over-recruit at the initial stage, to allow for the likelihood that some participants will be lost to follow up (Thomson and Holland, 2003). Data collection therefore continued past the point where data saturation (Saunders et al., 2018) was reached in these first interviews, to allow for subsequent drop out and ensure demographic variation.

### Data analysis

Inductive thematic analysis was carried out in parallel with ongoing data collection. Interview transcripts were checked against the audio-recordings, and read and reread for familiarity. Data were coded with descriptive, versus and structural codes (Saldana, 2015), using NVIVO software. Codes were refined and combined iteratively as data collection continued, and themes describing manifest content were developed within the three subject areas derived from the research aims. The technique of constant comparison (Glaser and Strauss, 1967) was used to reconsider early analysis in the light of subsequent interviews and the differences between real and ideal expectations. To increase the validity of the analysis, one researcher analysed all the transcripts and another analysed a subset (10% of total); codes and themes were discussed and agreed. All transcripts were re-read to check and confirm the final thematic structure. Throughout the process of data collection and analysis, the researchers worked with a reflexive awareness of their own perspectives on the transition to motherhood and postnatal care, including professional knowledge and diverse personal experiences.

## Findings

Forty women participated in interviews, at gestations between 27 and 40 weeks (median 38 weeks). Thirty were recruited online and ten through face-to-face contact. Thirty two interviews were by telephone and eight were face-to-face at home or in a community location. Interviews ranged in length from 12 to 75 min (mean 31 min). Table 1 provides socio-demographic information on the interview participants. A further four women who initially agreed to participate were not interviewed: one changed her mind and three gave birth before they could be interviewed.

**Table 1:** Socio-demographic characteristics of interview participants (*n* = 40).

	**Number of women (%)**

**Age in years**	
Under 20	7 (17.5)
20–24	2 (5.0)
25–29	10 (25.0)
30–34	12 (30.0)
35+	9 (22.5)
**Ethnicity**	
Asian	3 (7.5)
Black	2 (5.0)
Mixed/multiple heritage	4 (10.0)
White	31 (77.5)
**Country of birth**	
UK	36 (90.0)
Outside UK	4 (10.0)
**Relationship status**	
With husband/partner	37 (92.5)
Single	3 (7.5)
**Physical or mental health condition**	
No	32 (80.0)
Yes	8 (20.0)
Postcode classification*	
Quintile 1 (most deprived)	6 (15.0)
Quintile 2	7 (17.5)
Quintile 3	13 (32.5)
Quintile 4	7 (17.5)
Quintile 5 (least deprived)	7 (17.5)
**Urban/rural location**	
City/large town	32 (80.0)
Village/countryside	8 (20.0)
**Planned place of birth**	
Birth centre	20 (50.0)
Hospital labour ward	19 (47.5)
Home	1 (2.5)
**Gestational age at interview**	
27–32 weeks	6 (15.0)
33–36 weeks	6 (15.0)
37–40 weeks	28 (70.0)

⁎ Using the Index of Multiple Deprivation, (Ministry of Housing Communities and Local Government, 2015).

**Fig. 1:**
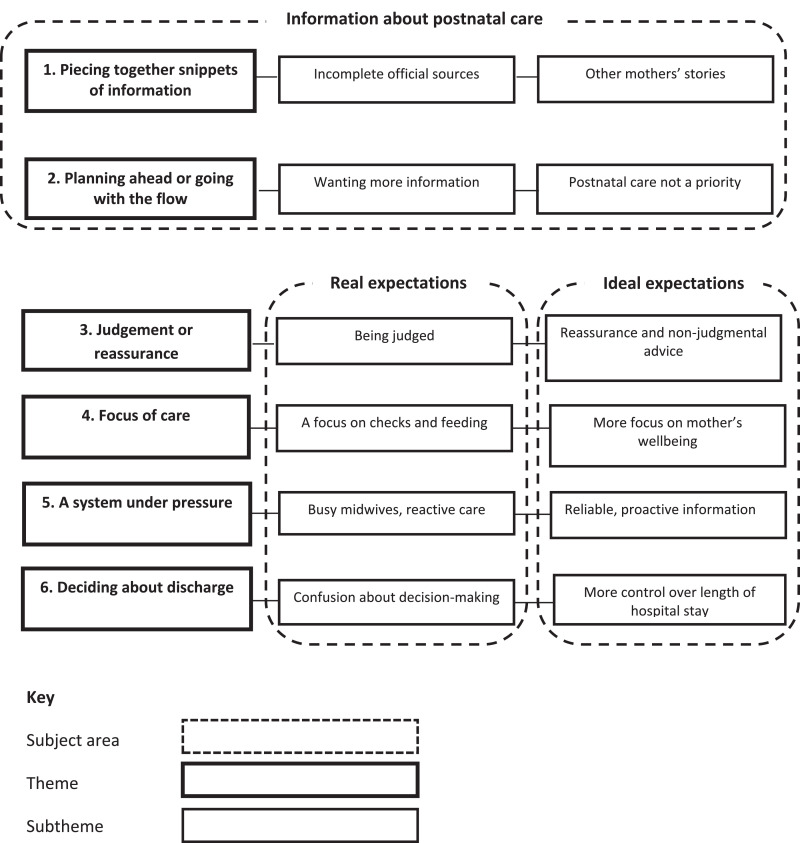
Antenatal expectations and information about postnatal care.

Some mothers had entirely unformed expectations about postnatal care, but most had some ideas and some had quite detailed expectations. Most distinguished between their real and ideal expectations: “*I think what I'd like it to be like is probably not what it's going to be”* (PNC055). There were no differences identified according to participants’ ethnicity, urban/rural location, relationship status or postcode quintile. Differences according to mothers’ age are described within the individual themes. The three subject areas, six themes and twelve subthemes are shown in Fig. 1.

## Piecing together snippets of information

### Incomplete official sources

None of the women had received comprehensive information about postnatal care. Those who sought it out said they had assembled “*snippets of information”* (PNC158), which were “*gleaned”* (PNC603), from different sources. Mothers noted that in antenatal appointments and classes only sketchy information on postnatal care was provided, unless the mother pressed for more: *“There wasn't lots of talk on what happens afterwards … They were happy to answer questions on it, but it wasn't a main focus”* (PNC192). Moreover, midwives who discussed midwifery postnatal care did not always explain how this fitted with other aspects of community postnatal care, such as the role of the health visitor, and vice versa: *“I don't know if that person is a midwife. I don't know how often they would visit me or why they would visit me*” (PNC091). One mother described how this fragmentation applied to all aspects of planning for postnatal life:

*“It's like when you get lost in the forest and you put down little bits of gingerbread to find your way out … the way that the NHS are offering [information], they've left you little bits of gingerbread to get out of the forest”* (PNC184).

### Other mothers’ stories

Lacking clear official information, women's expectations were instead largely based on the experiences and opinions of other mothers: *“friends and family and Googling”* (PNC110). This created uncertainty: *“Everyone's got a different view on what's going to happen… you pick pieces out of everyone's story”* (PNC501). Where these experiences were recent and local, they were very influential. They could create negative expectations: *“I've heard horror stories of people coming in and pretty much forcing you [to breastfeed]”* (PNC125); or positive ones: *“I've spoken to other people that have given birth round there… [the hospital midwives] are really good”* (PNC507). Women had found it particularly helpful to get mother-to-mother advance warning about practical issues such as recommendations to bring in flip flops for dirty showers and food for partners, as well as warnings not to take negative staff attitudes personally: *“And obviously that's not stuff you're going to find out, is it, from the NHS?”* (PNC192).

## Planning ahead or going with the flow

### Wanting more information

The majority of women expressed the wish to have more official antenatal information about postnatal care then they had actually received: “*I'm a natural planner, so I like to be forewarned before being in a situation”* (PNC129). Some primarily wanted a schedule of postnatal contacts with all professionals, similar to the personal maternity record. Others wanted to be reassured about the type of support available: “*I didn't have any concept of what happens afterwards* … *If I knew what postnatal care we'd be having, then it would help*” (PNC062). Some specifically noted that this would help them feel less stressed in facing the unknown scenario of the transition to motherhood: *“more information so when it happens, I don't panic”* (PNC146). Lacking information, some mothers imagined postnatal care scenarios that were unlikely and alarming: “*One baby crying I could probably deal with, 30 or 40 is going to be a bit of a struggle”* (PNC507).

### Postnatal care not a priority

About a quarter of the women had the opposite opinion, and did not feel the need to have detailed information about postnatal care in advance: “*I'm that kind of person that goes with the flow”* (PNC240). Some said that this was because they were so focused on pregnancy and giving birth that (even at 40 weeks) they had not thought about what would happen afterwards and did not want to: *“There is so much to think about right now in terms of antenatal care and giving birth, that the postnatal care, we're just going to have to see how it goes and ask for help at the time, if we need it”* (PNC046).

## Judgement or reassurance

This was the strongest theme for both real and ideal expectations.

### Real expectation: being judged

Women of all ages feared that they would be judged or patronised by professionals, because of their inexperience of motherhood: *“Someone that comes in and seems all official and stuffy, and then you feel like you're being judged for asking a silly question”* (PNC152). For the youngest mothers this expectation could be intensified by previous experiences of age discrimination: *“It's a bit patronising when they're doing it as if you're a six or seven-year-old, and you're not. I'm a young adult”* (PNC513, aged 17). Some of the young mothers were facing a parenting assessment after birth to determine whether their babies could live with them, and this shaped their perception of postnatal care as a process of scrutiny: *“[The midwife] has to come and … check on everything that's going on. I'll also have a social worker coming to check if I'm doing everything okay… to make sure that I'm not harming [the baby]”* (PNC507, aged 17).

For older mothers, worries about being judged were closely linked to their own apprehensions about feeling out of their depth with parenting: *“The emphasis in my generation, was ‘Go to school, go to university, get a master's degree,’ so now I'm 35 and I'm like, ‘Oh gosh, I'm about to have a baby, and I don't know how to do this’*” (PNC131).

In particular, they contrasted this expectation with their experience of being capable and respected in their working lives: *“As an adult, you get well used to being in charge of your life and calling the shots … you've never had to be looked after or told what to do, and then suddenly you have to relinquish all of that”* (PNC184, aged 35).

### Ideal expectation: reassurance and non-judgmental advice

The majority of participants described a desire for reassurance and accessible, non-judgemental advice to be at the heart of postnatal care. This ideal expectation was repeatedly described as a need to understand what was normal for babies and to have their own performance as a new mother affirmed: *“Reassurance that you're doing okay, or guidance if you're not … ‘This is what to expect, it's normal to feel like this, or it's not normal’*” (PNC046). It was paramount that this advice and reassurance should be given in a way that did not make the mother feel *“stupid”* (PNC062), but developed her parenting confidence: *“I want support in being independent”* (PNC272). Women also wanted professionals to acknowledge the specific challenges of first time parenthood:

*“Having someone that you can talk to who can allay your fears, because obviously it's been happening to women for hundreds of years, but it's the first time it's happened to me. You don't want to appear neurotic, but then at the same time you've got the weight of the world on your shoulders because you've got this new baby who's totally dependant on you.”* (PNC060)

## Focus of care

### Real expectations: a focus on checks and feeding

Most mothers expected physical checks on their baby and themselves to be the primary aim of postnatal care, and many thought the focus was likely to be more on the baby than the mother: *“You don't matter anymore, you get all this wonderful attention when you're pregnant, but I'm convinced that as soon as that baby comes out, it will be like, ‘Brilliant, now get to being a mum’”* (PNC184). Some also expected checks on their emotional wellbeing: *“Making sure that I'm coping okay, so I guess the mental side of things”* (PNC192). Almost all were planning to breastfeed and many expected that this might be difficult and would be another key part of postnatal care: *“Breastfeeding is what everyone has said, that's the main help you get”* (PNC224).

### Ideal expectations: more focus on the mother's wellbeing

Mothers said that they would ideally like to have more attention paid to the mother's wellbeing. For some, this was primarily about physical recovery: *“How has the birth been and are you suffering any ill effects … could it cause you any long-term problems?”* (PNC055). For others, it needed to include mental health and parenting confidence: *“Making sure that you're okay and making sure that you're ready to look after this baby for the rest of its life”* (PNC105). Mothers wanted to know that someone would be checking how they were feeling, and wanted to be offered support, guidance on emotional self-help, and suggestions for how their partners could help if they were struggling.

## A system under pressure

### Real expectation: busy midwives, reactive care

Mothers who had visited birth centres had all formed a positive impression of their calmness and comfort as places to stay. By contrast many expected postnatal wards to be busy and lacking in privacy: *“Everyone I've spoken to about staying overnight on the ward has said, ‘You just won't sleep because it's so noisy’”* (PNC131). Many said that they believed maternity hospitals were *“overstretched”* (PNC702) and some foresaw that this would have implications for personalisation of care on a postnatal ward: *“[Midwives are] going to have their checklist of things that they need to do, and are not going to be necessarily having the time to spend ages checking on my wellbeing”* (PNC012).

Many mothers said that they expected postnatal support to be reactive, with the onus being on the parents to ask for help if required: *“They're not going to be actively coming out, ringing you or checking to make sure you're okay”* (PNC125). For a few mothers this was a positive, empowering expectation: *“Being able to go off and do your own thing but knowing that there is the support”* (PNC250). Others said they would feel inhibited from asking for help that was not specifically offered: *“I won't ever ask for help, because I don't want to be in the way”* (PNC189), particularly when midwives were so busy, because they were worried about being seen as *“wasting their time”* (PNC060).

### Ideal expectation: reliable, proactive information

Women expressed a desire for the National Health Service (NHS) to provide clear, authoritative, consistent information about looking after a baby: *“That place to ring or go to, that isn't Google - which tells you that everything's going to be awful and your baby's going to die if they've got a little spot”* (PNC110). Several pointed out that whereas a new mother might not even know what questions she needed to ask, midwives would be aware of the typical issues and could ideally give this information proactively: *“A booklet where everything is there… they are dealing with this on a daily basis, so they might know … what the most important questions are”* (PNC704). They stressed that both parents needed to be included when information was being given or skills were being taught, and suggested that as well as supporting the partner's own transition to parenthood, partner-inclusive care would help the mother when postnatal care was under-resourced: *“The NHS is running out of money, if you've got a very supportive partner, they're a very good resource that could be utilised a little bit more to make women feel a lot more supported”* (PNC194).

## Deciding about discharge

### Real expectation: confusion about decision-making

Most women had heard about policies of prompt postnatal discharge, and some saw these as related to capacity issues: *“They need to make people go home as well, in order to cater for other people”* (PNC102). Some believed that irrespective of capacity, there would be pressure to leave quickly if the birth went well: *“They don't actually want you to stay there…they try and basically boot you out hours afterwards”* (PNC513). Their assumption was that health professionals had the authority to decide this: “*the doctor's sole decision*” (PNC161), and some were worried that this might mean they would be asked to leave before they felt ready: *“I don't want to be rushed out of hospital, because I want to make sure that everything's fine before you leave”* (PNC151)*.* Some had understood that even if staff were ready to discharge them, they could ask to stay if they were not feeling confident: *“There's a little bit of negotiation room”* (PNC194). Others said that they would be too diffident to challenge staff opinions: *“If a doctor or the midwife thinks the baby and I are fine to go home, I wouldn't really feel as though I had much power to argue with them”* (PNC046).

A second group worried that they might be made to stay in hospital longer than they wanted, and expected to have to pass a series of tests before being given permission to leave by health professionals: *“Obviously they won't let you leave the hospital until they know that you are able to feed, that you are able to do basic things like going to the toilet”* (PNC158). One had specifically asked her midwife whether she could decide to discharge herself, and she found the midwife's response was not reassuring: *“There certainly never has been a, ‘Yes or no, you have the power to leave when you want.’ They have the power to stop you … It is another factor that adds stress to the aftercare situation with midwives”* (PNC184).

A third group of women expected that discharge would be a joint decision, with a recommendation from staff followed by a decision by the parents: *“Mutual that I'm ready to go home and they also would advise in that area, ‘You are all fine and you can go’”* (PNC704). Finally a couple of mothers believed that discharge would be entirely their own decision: *“They say you can stay as long as you like”* (PNC270).

### Ideal expectation: more control over length of hospital stay

For those who were worried they would be discharged before they felt confident, their ideal was about having the choice to stay “*a little bit longer even if I was medically okay”* (PNC194). Likewise for those who were worried about being prevented from leaving hospital, there was the desire to be listened to so that any discharge decision would be *“a joint thing”* (PNC189).

## Discussion

This research demonstrates the anxieties that some pregnant women had about postnatal care, and their perception that a lack of information on which to base concrete expectations contributed to increasing their stress in the transition to parenthood. This absence of joined-up information is contrary to the national guidance that women should receive a personalised postnatal care plan while they are pregnant (Demott et al., 2006), and that care should be woman-centred rather than professional-centred (National Maternity Review, 2016). Instead women had to piece together a picture of what to expect and were largely reliant on what they could find out from informal sources such as other mothers’ stories, setting up a feedback loop where one mother's negative experiences could adversely affect other women's expectations. This reflects a wider concern amongst first time mothers of being ill-prepared for postnatal life in general and uncertain about which information to trust as they seek it out from multiple sources both online and offline (Aston et al., 2018; Entsieh and Hallstrom, 2016; Guerra-Reyes et al., 2017; Henshaw et al., 2018; Price et al., 2018). It has been argued by some health professionals that expectant parents are so focused on pregnancy and birth that they do not prioritise information about postnatal life (Renkert and Nutbeam, 2001). However, the majority of women in this study expressed a wish to have more information during pregnancy, from a clear and authoritative source, about what postnatal care they could expect, from whom, when and with what purpose. The fact that this was not a priority for all emphasises the importance of individualised care (National Maternity Review, 2016).

By asking first time pregnant women about both their real and their ideal expectations of postnatal care and comparing these different types of expectations, this study has been able to identify the specific fears that some had about postnatal care failing to meet their needs and potentially bringing them into conflict with health professionals. Previous work on *real* expectations has highlighted mothers’ practical concerns (Beake et al., 2010; Bowling et al., 2012; Forster et al., 2008; Puthussery et al., 2010). Although mothers in this study had similar real expectations about the likely busyness and noise of the ward environment, their greatest concern was the expectation of being adversely judged on their performance as mothers. There was also a widespread real expectation that they would have to take the initiative in asking for help, suggesting that knowledge about this aspect of care has increased since Ockleford et al. (2004) observed a mismatch between women ’s expectations and reality on this point. The diffidence some mothers expressed about asking for help if they needed it is important in the context of the reduced numbers of routine postnatal community midwifery visits to new mothers, with visits now more likely to be determined by service pressures than by mothers’ needs (Demott et al., 2006; Royal College of Midwives, 2014). Real expectations about discharge from hospital were more complex than those reported in earlier studies (Forster et al., 2008; Hirst and Hewison, 2002; Lindberg et al., 2008), with many women voicing concerns about who had the power to decide and the extent to which decisions would be based on individual need or choice. As suggested by Lindberg et al. (2008), their ideal expectations about having a choice over length of stay may have reflected a desire for maintaining control in the uncertain postnatal period.

Pregnant women's *ideal* expectations have also previously been reported to focus on the practical aspects of postnatal care (Forster et al., 2008; Hirst and Hewison, 2002; Lindberg et al., 2008). However the strongest ideal expectation for the majority of women in this study related to staff attitudes and their own confidence: the desire to be sure of ready access to non-judgmental advice, reassurance and affirmation from health professionals who would be kind and empathetic about the unique vulnerability of the first time mother. This was true for mothers of all backgrounds and all ages, including older mothers worried about losing status and role identity and younger mothers worried about age discrimination and child protection proceedings. The prominence of this desire closely reflects empirical findings that the manner in which postnatal information and advice are given are experienced as equally important as the content, and new mothers are highly sensitive to feeling judged (Aston et al., 2018; Henshaw et al., 2018; Lagan et al., 2014; Leahy Warren, 2005; Roche et al., 2005; Schmied et al., 2011; Wiklund et al., 2018).

This is the first qualitative research to explore in depth first time mothers’ expectations and information needs about postnatal care prospectively, distinguishing real and ideal expectations. It was a strength of this research that by combining initial recruitment through a survey with recruitment by face to face invitation, it included mothers who were diverse in age and socio-economic position. In particular it over-represented mothers aged under 20 - 17.5% of participants, compared with 2.5% of first time mothers giving birth in England (Office for National Statistics, 2019) - who are least likely to respond to surveys about maternity care. Participants were also diverse in their geographical location within England, so the findings do not relate to any specific hospital or area. It was a limitation that, despite efforts to reassure participants about interview confidentiality, some young women talked about their positive expectations of postnatal care, and their enthusiasm to make use of all help available, in terms that suggested they may have been trying to demonstrate an attitude of compliance with professionals. In addition, there was a wide range of length of interviews (12–75 min), reflecting the considerable variation in the extent to which participants had formed expectations about postnatal care. Three of the interviews were unusually short (15 min or less), limiting their depth: these participants had unformed expectations and found it challenging to engage with questions about what might happen in the future. Future research could explore the potential for local and national sources of postnatal care information to be available for pregnant women, and women's preferred formats. It could also consider fathers’ and co-parents’ expectations and information needs about postnatal care.

## Conclusions

This study identified how first time mothers need antenatal access to clear and comprehensive information about the content and purpose of postnatal care, as a standard part of their preparation for parenthood, so they can form realistic expectations. Information should take a woman-centred perspective and cover both hospital/birth centre and home/community, including the roles and responsibilities of all the professionals who may be involved. It should specifically address women's fears about postnatal care, in particular their worries about being judged as neurotic and incompetent if they ask questions and seek reassurance. Since not all women will engage with this information antenatally, it should be provided in ways that are accessible at any stage of pregnancy or the postnatal period.

## Author contributions

Jenny McLeish:  Methodology, Investigation, Formal analysis, Writing – Original Draft. Merryl Harvey: Methodology, Investigation.  Maggie Redshaw: Conceptualization, Methodology, Writing – Review and Editing. Fiona Alderdice: Conceptualization, Methodology, Formal Analysis, Writing – Review and Editing.
